# The tumor distance to the main hepatic vessels is a predictor of recurrence-free survival and overall survival in hepatocellular cancer

**DOI:** 10.1007/s00423-024-03565-9

**Published:** 2025-01-09

**Authors:** Schaima Abdelhadi, Johann S. Rink, Matthias F. Froelich, Flavius Șandra-Petrescu, Mohamad El-Ahmar, Hani Oweira, Nuh N. Rahbari, Christoph Reissfelder, Emrullah Birgin

**Affiliations:** 1 Department of Surgery, Medical Faculty Mannheim, Universitätsmedizin Mannheim, Heidelberg University, Theodor-Kutzer-Ufer 1-3, 68167 Mannheim, Germany; 2 Department of Radiology and Nuclear Medicine, University Medical Centre Mannheim, University of Heidelberg, Mannheim, Germany; 3 Department of General and Visceral Surgery, University Hospital Ulm, Ulm, Germany; 4 DKFZ-Hector Cancer Institute, Medical Faculty Mannheim, Heidelberg University, Mannheim, Germany

**Keywords:** Hepatocellular carcinoma, Hepatectomy, Radiology, Biomarker, Perioperative oncology

## Abstract

**Introduction:**

The impact of the distance of the tumor from the main hepatic vessels (DTV), such as the Glissonean pedicle or hepatic veins, on oncological outcomes for Hepatocellular carcinoma (HCC) patients is relatively understudied. Therefore, the objective of this study was to explore the correlation between DTV and survival in patients with HCC after curative hepatic resection.

**Methods:**

Consecutive patients who underwent curative-intent liver surgery for HCC between April 2018 and May 2023 were identified from a prospective database. Univariate and multivariate Cox regression analysis were performed to identify independent predictors of recurrence-free survival (RFS). A ROC-curve was used to find the optimal cut-off value for DTV. According to the estimated cut-off value, patients were divided into 2 subgroups, then using the Kaplan-Meier survival curve, RFS and overall survival (OS) were estimated and compared between the 2 subgroups.

**Results:**

In univariate analysis, DTV, tumor size, resection margins, microvascular invasion (MVI) and tumor grading were associated with RFS. In multivariate analysis, DTV, tumor size, and MVI were confirmed as independent predictors of RFS. In the ROC-analysis the optimal cutoff value of DTV was 20 mm. Patients with a DTV < 20 mm had a larger tumor size and a more advanced histopathological grading. There was no difference in the presence of MVI in both groups, while a significantly more patients experienced recurrence after hepatectomy in the DTV < 20 mm group. Accordingly, patients with a DTV < 20 mm experienced a shorter median RFS and OS.

**Conclusion:**

DTV is a promising predictor of RFS and OS in HCC.

**Supplementary Information:**

The online version contains supplementary material available at 10.1007/s00423-024-03565-9.

## Introduction

Hepatocellular carcinoma (HCC) ranks as the sixth most prevalent malignancy globally, with a notable rise in incidence [1]. It stands among the primary causes of cancer-related deaths, contributing to nearly 600.000 deaths annually [1]. Notably, within Europe, the emergence of HCC in non-cirrhotic livers is increasingly observed [2]. Liver resection, apart from liver transplantation, remains the pivotal approach for achieving curative treatment. Over time, substantial progress in operative techniques and perioperative care has led to a reduction in postoperative morbidity and mortality. Nevertheless, long-term outcomes remain unsatisfactory due to a substantial recurrence rate, reaching up to 70% within 5 years [3]. Various predictive factors for HCC recurrence have undergone extensively scrutiny, yielding insights into disease trajectories. Variables such as tumor size [4, 5], multifocality [6], microvascular invasion (MVI) [5, 7] poor histological differentiation [5] and compromised tumor capsule integrity [4, 7] independently constitute risk factors for early HCC recurrence. However, a relatively understudied aspect is the impact of the distance of the tumor from the main hepatic vessels (DTV), such as the Glissonean pedicle or main hepatic veins, on oncological outcomes. Given that the infiltration of major hepatic vessels is associated with advanced disease and poor survival, we hypothesized that the proximity of HCC lesions to major hepatic vessels could also advocate poor outcome owing to an increase of local angiokines with higher vascularization [8, 9]. Therefore, the objective of this study was to explore the correlation between the distance of the tumor from the main hepatic vessels and survival in patients with hepatocellular cancer after curative hepatic resection.

## Methods

### Study design and patient cohort

**Fig. 1:**
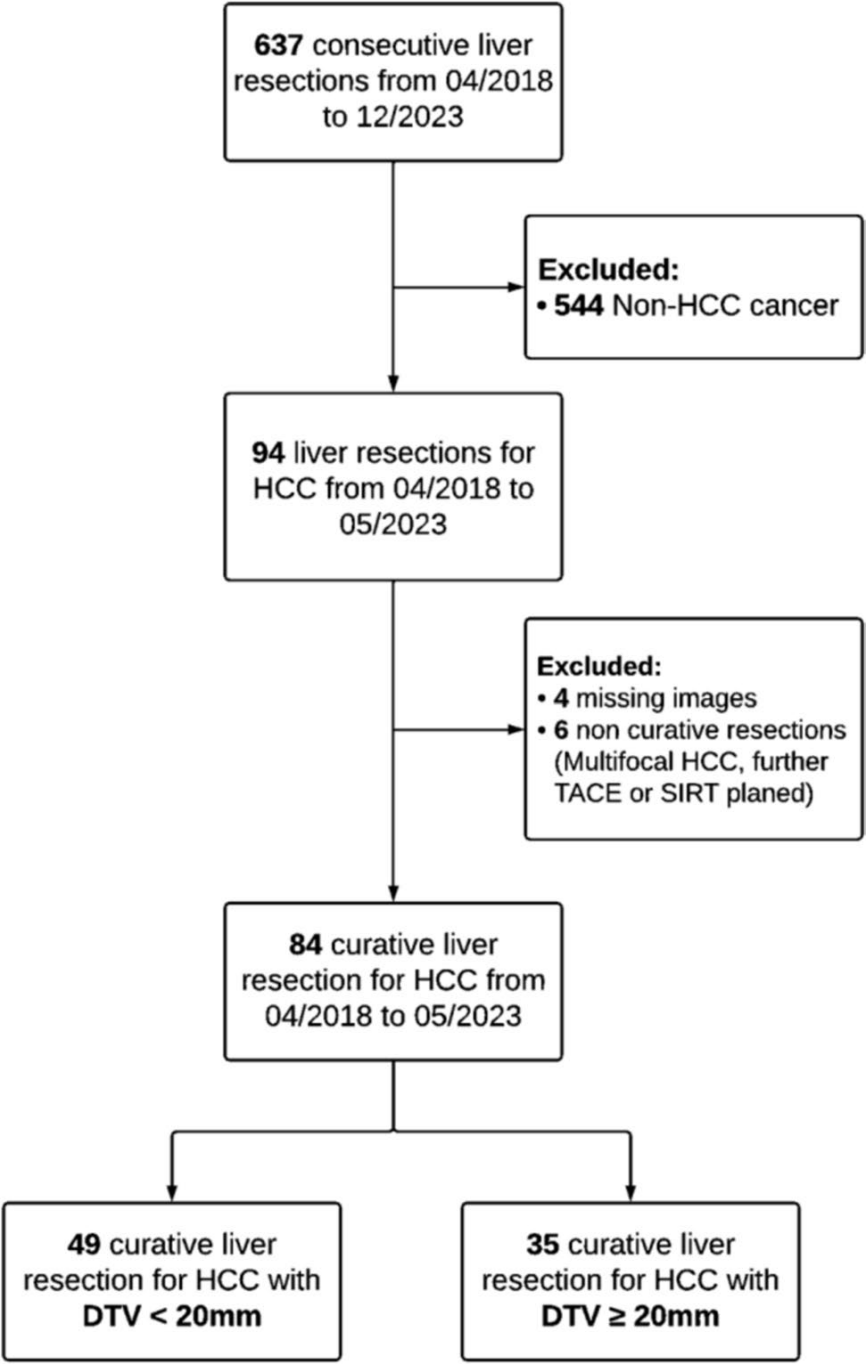
Patient flow chart

All consecutive patients who underwent liver surgery for HCC between April 2018 and May 2023 were identified from a prospectively collected institutional database at the Department of Surgery, University Hospital Mannheim, Heidelberg University [10–12]. Patients were eligible for inclusion if they were aged 18 years or older, underwent a curative-intent liver resection for HCC, and had adequate preoperative imaging, including contrast-enhanced computed tomography (CT) and/or magnetic resonance imaging (MRI). Patients with multifocal HCC with further SIRT or TACE planned were excluded. Additionally, we excluded patients who had inadequate quality or missing preoperative imaging (Fig. 1). This cohort study was conducted in line with the STROBE guidelines and approved by the ethics committee at Heidelberg University (2023 − 831) [13].

### Definitions and data acquisition

We extracted the following demographic and preoperative historical data, including medical comorbidities and laboratory values: age, gender, body mass index (BMI), American Society of Anesthesiologists (ASA) score classification, cardiovascular comorbidities, pulmonary comorbidities, diabetes mellitus, presence of liver cirrhosis, Child-Pugh classification, underlying liver disease, preoperative treatment, preoperative laboratory values such as, albumin, bilirubin, INR (International Normalized Ratio), platelet count, alkaline phosphatase, gamma-glutamyltransferase, aspartate-aminotransferase and alanine-aminotransferase.

Further data assessed included intra- and postoperative details, such as extent of resection, the use Pringle maneuver, duration of Pringle maneuver, the use of infrahepatic vena cava (IVC) clamping, operative time, blood loss, pathological data, postoperative length of stay and postoperative complications. Pathological data included the tumor size, resection margin and presence of MVI. Postoperative complications were graded according to the Clavien-Dindo classification. Specific complications after liver resections were classified and reported following the recommendations of the International Study Group of Liver Surgery [14, 15].

Patients were deemed suitable for curative resection if they had resectable HCC lesions with adequate future liver remnant, liver function, performance status, the absence of distant metastasis or portal vein thrombosis as described previously [10–12, 16]. The Brisbane nomenclature was used to classify liver resections [17]. Anatomic liver resections were defined in line with Couinaud’s portal segmentation and the complete removal of a portal territory with its corresponding parenchyma [17].

Dates of last follow-up, recurrence, and death were recorded to calculate recurrence-free survival (RFS) and overall survival (OS). RFS was defined as the time from date of curative surgery to the time of recurrence (radiologic or histologic evidence of local, regional, or distant metastasis) or death by any cause, while OS was defined as the time from date of curative surgery to the time of death.

### Standardization of perioperative care

All patients received pre-, intra- and postoperative care according to the local standard protocols established within a well-defined multidisciplinary framework [10–12]. The indications for resection in suspected HCC were discussed preoperatively in multidisciplinary tumor board conferences. All surgeries were performed by experienced attending hepatobiliary surgeons. Lymphadenectomy was not routinely performed; only in cases where enlarged or suspicious lymph nodes were found.

### Imaging analysis

**Fig. 2:**
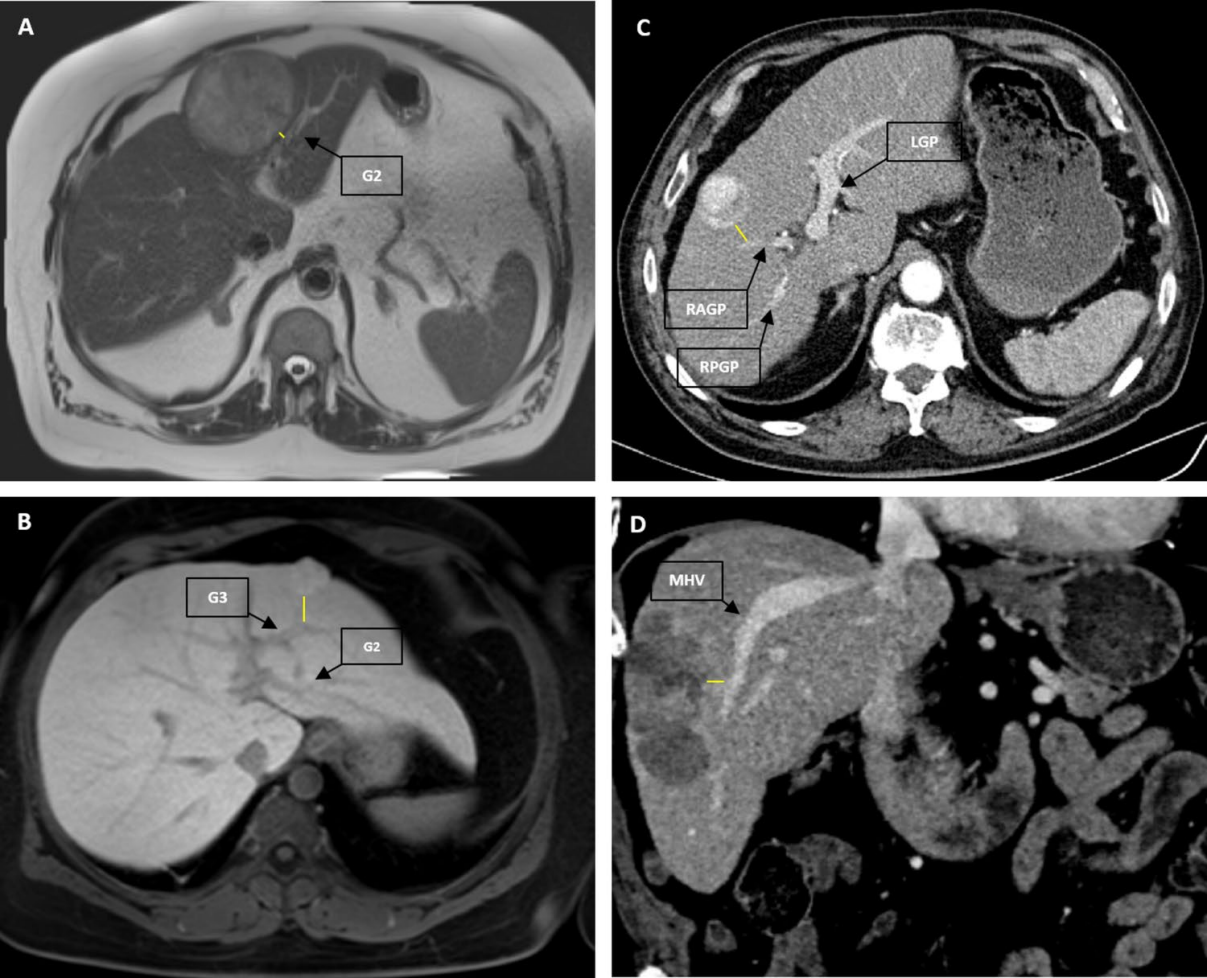
DTV-Measurement based on preoperative Imaging. Hepatic preoperative MRI (A-B) and CT (C-D). A: T2-weighted, B: Fat saturated T1-weighted. C: Arterial phase, D: Portal venous phase. DTV (yellow line) measured at the point of nearest distance between the tumor and any of the main hepatic vessels, such as the Glissonean pedicle (first- and second-order branches) or hepatic vein at caval confluence. *G2* Glissonean pedicle of segment 2; *G3* Glissonean pedicle of segment 3; *LGP* left Glissonean pedicle; *RAGP* right anterior Glissonean pedicle; *RPGP* right posterior Glissonean pedicle; *MHV* middle hepatic vein

Preoperative CT and MRI images were independently reviewed by two radiologists (JR, MF), who were blinded to the clinical, surgical, pathologic, and follow-up results. DTV was defined as the nearest distance between the tumor and any of the main hepatic vessels, such as the Glissonean pedicle (first- and second-order branches) or main hepatic veins, including the main trunk of the right, middle and left haptic vein (Fig. 2). If there where multiple tumor lesions, DTV was measured based on the largest lesion. If the lesions were of a similar size, the shortest DTV was measured.

### Statistical analysis

The statistical analysis was performed using R version 3.6.5. Categorical parameters are expressed as frequencies and were compared using the Pearson χ2 test or Fisher exact test. Continuous variables are reported as mean (SD) or median (IQR), depending on the distribution pattern, and were compared using the two-tailed t test or Mann-Whitney test. Univariate and multivariate Cox proportional hazards regression model were performed to identify independent predictors of RFS.

A receiver operating characteristic (ROC) curve and relative area under the curve statistics were used to find the optimal cut-off value for DTV. The continuous variable DTV was applied to ROC analysis with the endpoint of tumor recurrence and mortality. According to the estimated cut-off value, the patients were divided into 2 subgroups, then using the Kaplan-Meier survival curve RFS and OS were estimated and compared between the 2 subgroups and the log-rank test was used to analyze statistical significance of Kaplan–Meier estimates. All p-values were considered statistically significant when the association probability was less than 0.05.

## Results

A total of 637 consecutive liver resections were recorded in the database, of which 544 resections were excluded due to non-HCC cancer, resulting in a total of 94 liver resections during the study period. Following the exclusion of 4 cases with inadequate or missing preoperative imaging and 6 cases with non-curative liver resections, the final study cohort comprised 84 patients. The baseline and operative characteristics, as well as the postoperative and oncological outcomes of the study cohort are detailed in Table 1 and S1. Among the study population, 29 (35%) patients experienced HCC recurrence after a median follow-up of 19 months (IQR: 3–33).

**Table 1:** Baseline characteristics of the study population

Characteristics	Total (*n* = 84)
Age (years) ^a^	70 (63–78)
BMI (kg/m²) ^a^	27 (24–29)
Sex ratio (Male: Female)	67:17
ASA	
I	2 (2)
II	36 (43)
III	45 (54)
IV	1 (1)
Cardiovascular comorbidities	67 (80)
Diabetes mellitus	32 (38)
Pulmonary comorbidities	26 (31)
Liver cirrhosis	34 (40)
Child-Pugh classification	
Child A	24 (28)
Child B	10 (12)
Etiology of cirrhosis	
Alcohol	18 (21)
Viral	12 (14)
Hepatitis B	4 (5)
Hepatitis C	8 (10)
MASLD	4 (5)
Preoperative laboratory tests ^b^	
Albumin (g/l)	36 (6)
Bilirubin (mg/dl)	0.7 (0.4)
INR	1.1 (0.1)
Platelets (× 10^9^/l)	234 (109)
AP (U/l)	127 (117)
gGT (U/l)	170 (206)
AST (U/l)	52 (73)
ALT (U/l)	47 (48)
Previous treatment	
Previous hepatic resection	15 (18)
Previous locoregional therapy	3 (4)
Previous Y90 treatment	2 (2)
Previous systemic treatment	2 (2)
Radiological characteristics ^a^	
DTV, mm	18 (7–26)
Number of lesions	1 (1–2)
Tumor size, mm	50 (25–70)
Number of infiltrated segments	2 (1–3)

*BMI* body mass index, *ASA* American Society of Anesthesiologists, *MASLD* metabolic dysfunction-associated steatotic liver disease, *INR* international normalized ratio, *AP* alkaline phosphatase, *gGT* gamma-glutamyl-transferase, AST aspartate aminotransferase, *ALT* alanine aminotransferase, *DTV* distance of the tumor from the main hepatic vessels ^a^Values are median (interquartile range) ^b^Values are mean (standard deviation)

### Predictive factors for recurrence-free survival

To identify predictive factors for RFS, both univariate and multivariate analysis were performed using the Cox proportional hazard regression model (Table 2 and S2). In univariate analysis, DTV (HR 0.91; 95%CI 0.87–0.95; *p* < 0.001), tumor size (HR 1.01; 95%CI 1.00–1.02; *p* < 0.001), resection margin (HR 3.41; 95%CI 1.08–9.26; *p* = 0.03), tumor grading (HR 3.41; 95%CI 11.25–9.32; *p* = 0.017 in grade 2 and HR 11.5; 95%CI 3.24–40.79; *p* < 0.001 in grade 3) and MVI (HR 4.53; 95%CI 2.13–9.61; *p* < 0.001) were associated with RFS (Table S2). Variables with *p* < 0.05 were then subjected to further evaluation in a multivariate analysis to assess their predictive potential independent of other clinical characteristics. In the multivariate analysis, DTV (HR 0.92; 95%CI 0.89–0.99; *p* = 0.03), tumor size (HR 1.01; 95%CI 0.99–1.02; *p* = 0.02) and MVI (HR 2.99; 95%CI 1.18–7.55; *p* = 0.02) were confirmed as independent predictors of RFS (Table 2 and S2).

**Table 2:** Clinicopathological characteristics and outcomes of patients stratified by a DTV of 20 mm

Characteristics	DTV < 20 mm (*n* = 49)	DTV ≥ 20 mm (*n* = 35)	*p*-value
Age (years) ^a^	70 (66–72)	72 (68–75)	0.27
BMI (kg/m²) ^a^	26 (24–28)	28 (25–32)	0.19
Sex ratio (Male: Female)	40:9	27:8	0.62
ASA			0.14
I	1 (2)	1 (3)	
II	18 (38)	18 (51)	
III	29 (59)	16 (46)	
IV	1 (2)	0 (0)	
Cardiovascular comorbidities	40 (81)	27 (77)	0.62
Diabetes mellitus	19 (39)	13 (37)	0.88
Pulmonary comorbidities	11 (22)	15 (43)	0.06
Liver cirrhosis	22 (45)	14 (40)	0.33
Child-Pugh classification			
Child A	14 (28)	10 (28)	
Child B	8 (16)	2 (6)	
Etiology of cirrhosis			0.31
Alcohol	12 (24)	6 (17)	
Viral	7 (14)	5 (14)	
Hepatitis B	2 (4)	2 (6)	
Hepatitis C	5 (10)	3 (8)	
MASLD	1 (2)	3 (8)	
Preoperative laboratory tests ^b^			
Albumin (g/l)	35.2 (5.5)	36.2 (5.4)	0.43
Bilirubin (mg/dl)	0.7 (0.4)	0.6 (0.3)	0.56
INR	1.1 (0.1)	1.1 (0.1)	0.25
Platelets (× 10^9^/l)	234 (109)	235 (120)	0.44
AP (U/l)	119 (80)	138 (156)	0.54
gGT (U/l)	143 (116)	208 (287)	0.67
AST (U/l)	59 (92)	442 (31)	0.33
ALT (U/l)	46 (48)	50 (47)	0.73
Previous treatment			
Previous hepatic resection	6 (12)	9 (26)	0.11
Previous locoregional therapy	2 (4)	1 (3)	0.49
Previous Y90 treatment	1 (2)	1 (3)	0.81
Previous systemic treatment	1 (2)	1 (3)	0.81
Radiological characteristics ^a^			
DTV, mm	10 (5–17)	25 (20–34)	< 0.001
Number of lesions	1 (1–2)	1 (1–2)	0.96
Tumor size, mm	62 (31–76)	41 (12–60)	< 0.001
Number of infiltrated segments	2 (1–3)	2 (1–2)	0.98
Extent of resection			0.70
Major hepatectomy	13 (27)	8 (23)	
Minor hepatectomy	36 (73)	27 (77)	
Surgical approach			0.52
Open	7 (14)	5 (14)	
Laparoscopic	35 (71)	26 (74)	
Robotic	7 (14)	4 (11)	
Surgical procedure			0.11
Non-anatomic resections	7 (14)	3 (8)	
Right (extended) hepatectomy	5 (10)	5 (14)	
Left (extended) hepatectomy	9 (18)	2 (6)	
Left lateral sectionectomy	6 (12)	4 (11)	
Right posterior sectionectomy	0 (0)	4 (11)	
Other anatomical segmentectomy	22 (45)	17 (48)	
Operative time, min^a^	259 (183–369)	240 (177–323)	0.43
Pringle maneuver	28 (40)	23 (65)	
Duration, min^a^	40 (21–63)	37 (27–72)	
IVC clamping	2 (4)	3 (8)	
Blood loss, mla	600 (300–1500)	500 (250–1150)	0.35
Resections margins			0.67
R0	45 (92)	33 (94)	
R1	3 (6)	2 (6)	
T classification			0.73
1	22 (45)	19 (54)	
2	20 (41)	12 (34)	
3	5 (10)	4 (12)	
4	1 (2)	0 (0)	
X	1 (2)	0 (0)	
Nodal status			0.12
0	28 (57)	14 (10)	
1	0 (0)	0 (0)	
X	21 (43)	21 (60)	
M status			0.81
0	48 (98)	34 (97)	
1	1 (2)	1 (3)	
Grading			0.01
1	6 (12)	19 (54)	
2	29 (59)	14 (40)	
3	7 (14)	2 (6)	
X	7 (14)	0 (0)	
Microvascular invasion			0.08
0	34 (70)	29 (83)	
Recurrence status	22 (45)	7 (20)	0.02
Recurrence location			0.21
Lungs	7 (14)	0 (0)	
Liver (non-local)	10 (20)	4 (11)	
Local recurrence	5 (10)	3 (9)	
Length of stay, d^a^	8 (5–13)	7 (5–10)	0.42
Postoperative complications ^c^			0.21
Grade I	7 (14)	9 (26)	
Grade II	9 (18)	5 (14)	
Grade III	4 (8)	3 (8)	
Grade IV	1 (2)	4 (11)	
Grade V	5 (10)	1 (3)	
Type of complication			
Wound infection	3 (6)	2 (6)	1.00
Burst abdomen	2 (4)	0 (0)	0.26
Pleural effusion with atelectasis	6 (12)	7 (20)	0.38
Pulmonary embolism	0 (0)	2 (6)	0.89
Posthepatectomy hemorrhage	4 (8)	1 (3)	0.07
Posthepatectomy bile leakage	3 (6)	2 (9)	0.93
Posthepatectomy liver failure	6 (12)	4 (11)	0.91

*BMI* body mass index, *ASA* American Society of Anesthesiologists, *INR* international normalized ratio, *AP* alkaline phosphatase, *gGT* gamma-glutamyltransferase, *AST* aspartate aminotransferase, *ALT* alanine aminotransferase, *DTV* distance of the tumor from the main hepatic vessels, *IVC* infrahepatic vena cava, *MVI* microvascular invasion, *R0* no residual tumor, *R1* microscopic residual tumor, mth months, *X* missing data ^a^Values are median (interquartile range) ^b^Values are mean (standard deviation) ^c^Clavien-Dindo classification

### Analysis of a threshold for DTV

**Fig. 3:**
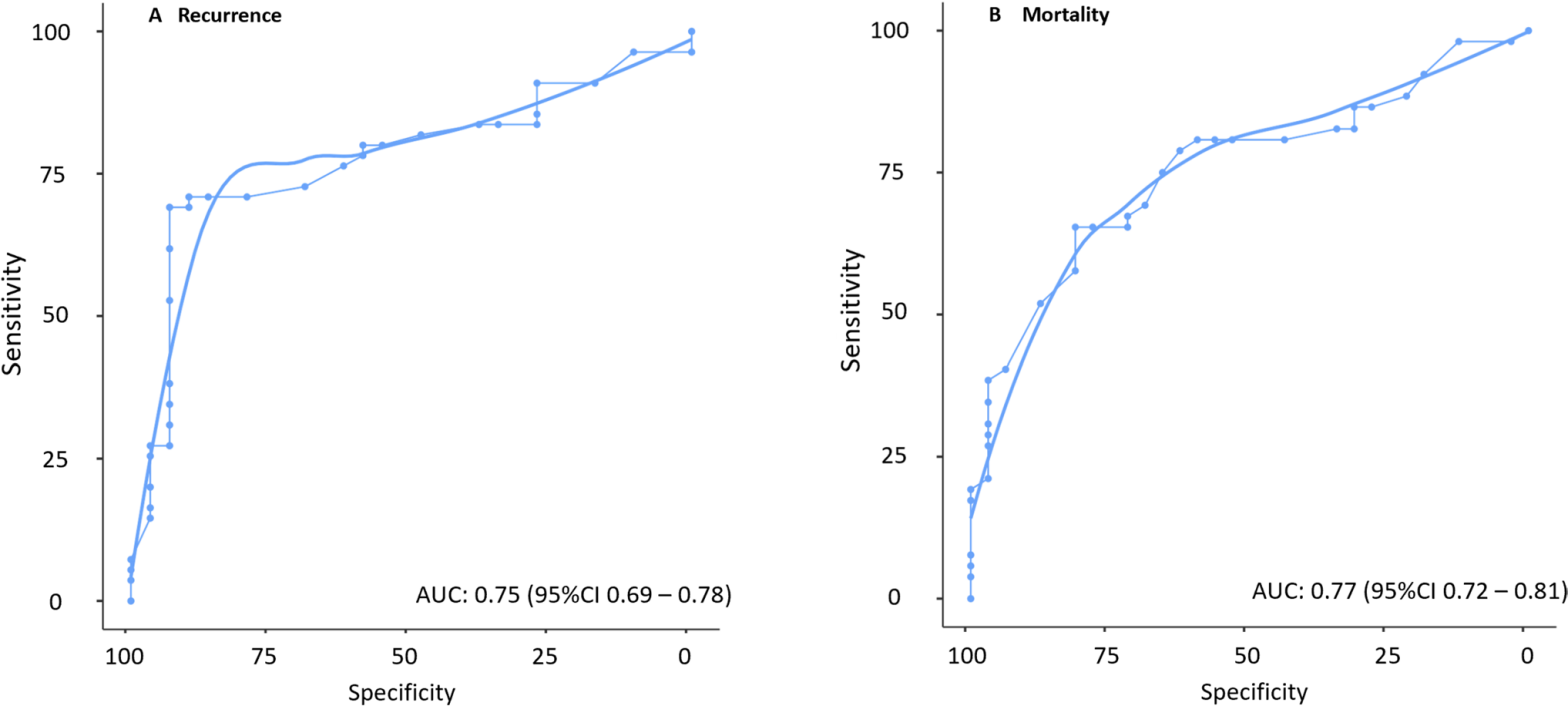
Receiver Operating Characteristics (ROC) curves for the optimal cutoff value of DTV

In a next step, the validity of DTV as a predictor for RFS and OS was assessed through ROC-Analysis. The optimal cutoff value of DTV was determined as 20 mm. This threshold predicted OS with a sensitivity of 65% and specificity of 81% (AUC: 0.75; 95%CI 0.69–0.78) (Fig. 3A) and RFS with a sensitivity of 69% and specificity of 93% (AUC: 0.77; 95%CI 0.72–0.81) (Fig. 3B).

### Stratified cohort analysis by a DTV of 20 mm

**Fig. 4:**
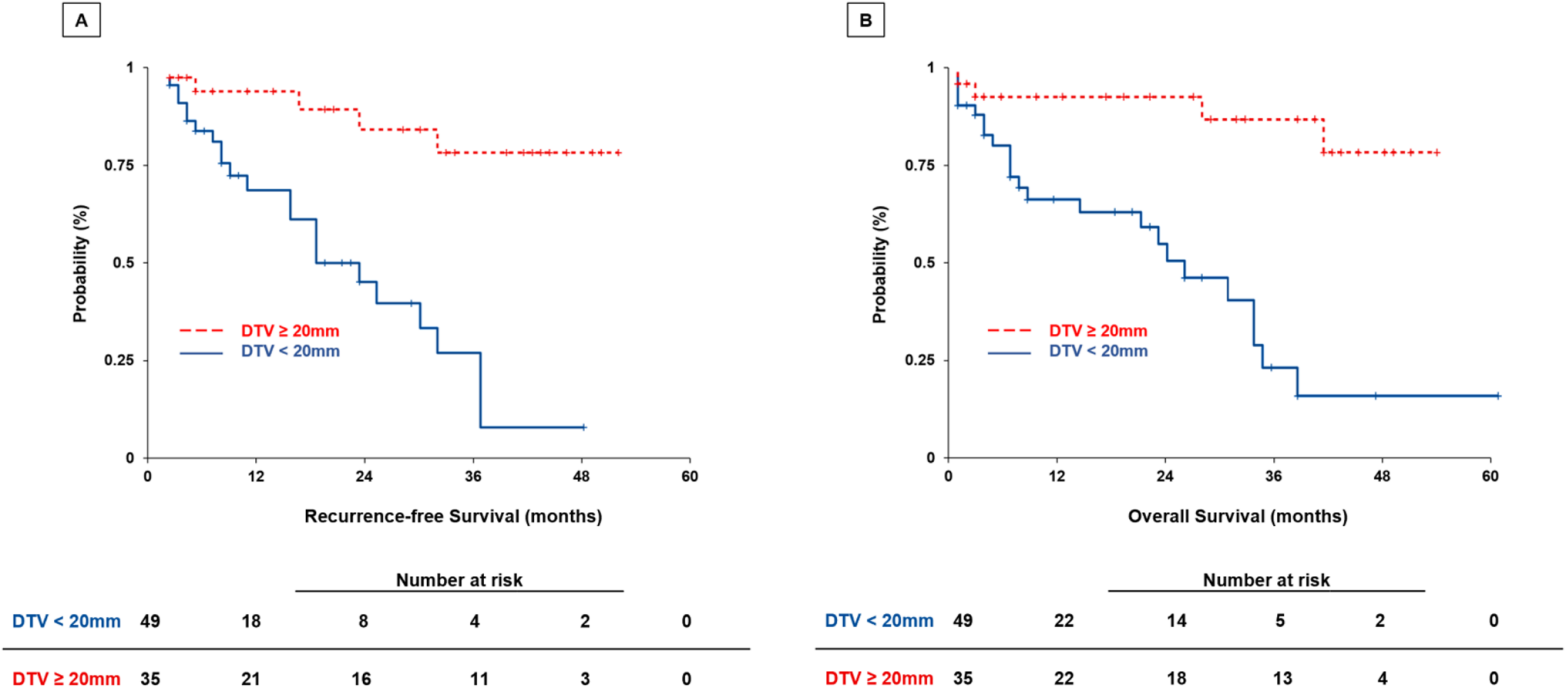
Survival plot stratified by 20 mm DTV

To further explore the clinicopathologic characteristics and outcomes based on the DTV, the cohort was stratified using a DTV of 20 mm (Fig. 1). Among the 84 patients, 49 (58%) had a DTV < 20 mm and 35 (42%) had a DTV ≥ 20 mm. Comparisons of baseline and operative characteristics and postoperative outcomes between the study groups yielded no statistical significance concerning age, gender, BMI, medical comorbidities, preoperative laboratory tests, previous treatment, extent of resection, surgical approach and procedure, operative time, blood loss, length of stay and postoperative complications (Table 3). Patients with a DTV < 20 mm had a larger median tumor size (47 mm, IQR: 35–68 vs. 30 mm, IQR 15–50, *p* < 0.001), and a more advanced histopathological tumor grading (G3: 14% vs. 6%; *p* = 0.01). There was no significant difference in the presence of MVI in both groups (30% vs. 17%, *p* = 0.08) (Table 3), while a significantly higher proportion of patients experienced recurrence after hepatectomy in the DTV < 20 mm group (45% vs. 20%; *p* = 0.02). In the DTV < 20 mm group, a total of 22 recurrences were observed, including 10 (20%) in the liver (non-local), 7 (14%) in the lungs, and 5 (10%) as local recurrences. In the DTV ≥ 20 mm group, a total of 7 recurrences (20%) were observed, including 4 (11%) in the liver (non-local) and 3 (9%) as local recurrences (*p* = 0.21). Accordingly, patients with a DTV < 20 mm experienced a shorter median RFS with 6 months (95%CI: 7–14) vs. 19 months (95%CI: 16–28; *p* = 0.003) as well as shorter median overall survival with 8 months (95%CI: 10–19) vs. 27 months (95%CI: 18–30; *p* = 0.002) (Fig. 4).

**Table 3:** Multivariate Cox proportional hazard analysis of predictive factors for recurrence-free survival

Variables	HR	95%CI	*p*-value
DTV, mm	0.94	0.89–0.99	***0.03***
Tumor size, mm	1.01	0.99–1.02	***0.02***
Resections margins (R1 vs. R0)	1.99	0.38–10.31	0.41
Tumor grading (G2 vs. G1)	2.17	0.75–6.28	0.15
Tumor grading (G3 vs. G1)	4.15	0.91–18.98	0.06
MVI (Yes vs. No)	2.99	1.18–7.55	***0.02***

*HR* hazard ratio, *CI* confidence interval, *DTV* distance of the tumor from the main hepatic vessels, *MVI* microvascular invasion, *R0* no residual tumor, *R1* microscopic residual tumor

## Discussion

To the best of our knowledge, this is the first study to evaluate the predictive value of DTV in HCC on RFS and OS based on preoperative imaging. Our results indicate that DTV is a significant predictor for RFS and OS in HCC after curative resection. A DTV ≥ 2 cm was associated with a high sensitivity and specificity for predicting RFS and OS for patients with HCC after curative resection. Moreover, the survival outcomes stratified by DTV achieved a significant difference of OS and RFS in patients with HCC.

The concept of “liver zonation”, established over two decades ago, highlights the importance of hepatocyte proximity to blood vessels in shaping their morphological and functional heterogeneity [18]. Despite the histological uniformity of liver tissue, heterogeneity exists in terms of morphometry and histochemistry [18]. At the histological level, the hepatic lobule represents the smallest anatomical unit, resembling a hexagonal structure with a central vein in the middle and portal triads at its corners, consisting of a branch from the portal vein, a branch from the hepatic artery and a bile duct [19]. Within the lobule hepatocytes are arranged in cord-like structures radiating from the central vein towards the portal triads, where they face blood channels called sinusoids at either side [19]. While lobules represent a more structural unit, the hepatic acinus is the functional unit of the liver and is divided into three zones based on proximity to the portal triads [19, 20]. Nutrient-rich blood from the gastrointestinal tract enters the sinusoid via the portal vein, mixes with oxygen-rich blood from the hepatic artery and flows through the sinusoids to the central vein [19, 20]. Due to metabolism and elimination along the hepatic sinusoids, the blood composition changes, resulting in gradients of oxygen, nutrients and hormones [18, 20–22]. These physical gradients establish a “zonation” of hepatocytes. Hepatocytes adjacent to the portal vein show high gluconeogenesis, urea synthesis, and beta oxidation activity. In turn, pericentral hepatocytes are characterized with increased glycolysis, bile synthesis, xenobiotics metabolism, and triglyceride synthesis [18, 20–22]. Consequently, it is reasonable to anticipate that liver cancer cells manifest different biological profiles based on their proximity to blood vessels. Aligned with this theory, previous studies have described different zonation profiles of glioblastoma cells based on their relative proximity to perfused blood vessels [8, 9]. In their study, Kumar et al. found that perivascular cancer cells displayed heightened metabolism, enhanced aggressiveness, and increased resistance to treatment [8, 9]. Consistent with these findings, our study demonstrates that HCC in closer proximity to the main hepatic blood vessels exhibits a significantly higher recurrence risk and mortality. Notably, these lesions also displayed a larger median tumor size and a higher tumor grading. These results suggest that proximity to the main hepatic blood vessels promotes tumor growths and aggressiveness, potentially attributed to varying exposure to nutrients and growth factors, as well as differential oxygen availability. Considering that HCC exhibits a high phenotypic and molecular heterogeneity, as well as the differences between the periportal and perivenous zone, there might be different underlying mechanisms leading to higher tumor growth higher tumor growth and aggressiveness in the each zone.

On one side, the periportal zone shows a higher activity of the hepatocyte growth factor (HGF) [23]. Several studies have demonstrated that HGF serves a significant role in the onset, proliferation, invasion, and metastasis of HCC [24–26]. One study has demonstrated that HGF induced invasion and migration of HCC cells [27]. These findings support the hypothesis that the proximity to the portal triads promotes tumor growths and aggressiveness in HCC. An additional factor might be the significant pO2 gradient depending on the proximity to the portal triads, ranging from approximately 60–65 mmHg (84–91 µmol/L) in periportal blood to about 30–35 mm Hg (42–49 µmol/L) in pericentral blood [18, 20–22]. Consequently, intracellular pO2 levels are lower by about 15 mmHg, measuring 45–50 mmHg in periportal cells and 15–20 mmHg in pericentral cells. The higher periportal oxygen availability may promote the enhanced tumor growths and aggressiveness in HCC in closer proximity to the portal triads. HCC is generally associated with hypervascularity and is dominantly supplied by arterial blood flow, with an arterial blood fraction of 50–60%, whereas the normal underlying liver parenchyma is supplied with an arterial blood fraction of 25% [29, 30]. This phenomenon suggests that HCC growth depends on a high oxygen availability, contradicting the commonly stated assertion that “hypoxia is a common characteristic of HCC” [28, 29]. In a critical evaluation published in 2021, Cramer et al. outlined the circumstances leading to the designation of human HCC as a “typically hypoxic tumor” and concluded that there is no reliable, published evidence supporting the existence of severe hypoxia in human HCC [28–30]. Up to this day, direct measurements of pO2 in human HCC have not yet been published, thus the functional relevance of hypoxia to hypervascularity and arterialization of HCC remains unsubstantiated [28–30].

Since oxygen constitutes the basis for formation of reactive oxygen species (ROS), ROS production is predominantly in the periportal zone [18]. ROS is a general term for highly unstable oxygen-containing derivatives produced by the partial reduction of molecular oxygen, such as hydroxyl radicals (•HO) and hydrogen peroxide (H2O2) [31]. In HCC, ROS can potentially enhance tumor cell proliferation, metastasis, and resistance to treatment [32]. These high ROS production in the periportal zone may promote tumor growths and aggressiveness in this zone.

On the other side, in the adult liver only perivenous hepatocytes express the Leucine-rich repeat-containing G-protein coupled receptor 5 (LGR5). LGR5 is crucial for tumor development and tumor cell signal transduction [33]. A recent study found that an upregulated expression of LGR5 was significantly correlated with larger tumor diameter (> 5 cm), higher TNM stage, recurrence and metastasis in HCC [34]. Moreover, it was found that in mouse livers Indian Hedgehog (Ihh) was expressed exclusively in the most distal perivenous area [20, 35]. Hedgehog signaling has a substantial role in hepatocarcinogenesis and HCC progression [36].

However, at present, the exact mechanisms leading to an enhanced tumor aggressiveness of HCC in closer proximity to the main hepatic blood vessels, remain largely unclear and require further investigation.

Apart from the enhanced tumor aggressiveness as a probable cause for the higher recurrence rate and significantly earlier tumor recurrence (6months vs. 19 months), the closer proximity to the main blood vessels means also a closer proximity to the lymphatic vessels, as lymphatic drainage patterns in the liver follow the segmental liver anatomy [37], which may enable the further spread of cancer cells to distant organs, which in turn, results in a higher recurrence and worse OS. While lymphatic capillaries along the portal triads drain to lymph nodes at the hepatic hilum and the lesser momentum, lymphatic vessels aside the hepatic veins traverse along the inferior vena through the diaphragm toward mediastinal lymph node [38]. According to the findings of recent studies, the most common location for extrahepatic metastasis from HCC include the lungs and the lymph nodes. Furthermore, it was found that HCC hematogenous spread mostly occurs via the lymphatic route or by direct vascular invasion [39]. These findings support the assumption, that the closer proximity to the main hepatic blood and lymphatic vessel may be a relevant factor for early spread of cancer cells and by this an early recurrence.

Despite the high recurrence rate of HCC, there is no effective adjuvant treatment recommended by the European Association for the Study of the Liver (EASL) or the American Association for the Study of Liver Diseases (AASLD) [3, 40]. Therefore, adequate knowledge of predictive factors to identify patients with a high recurrence risk is important for individualized treatment, management and surveillance strategies. Although there is no standard-of-care adjuvant treatment for patients with HCC, recent data from a randomized phase III trial have demonstrated the efficacy and safety of adjuvant atezolizumab plus bevacizumab in patients with HCC at high risk of recurrence following curative-intent resection [41]. Another randomized phase II trial demonstrated the effectiveness of the anti-PD-1 antibody sintilimab in patients with HCC with a high risk of disease recurrence due to MVI [42, 43]. Due to their higher recurrence risk, patients with HCC in closer proximity to the main hepatic blood vessels may also profit from these adjuvant treatment strategies. Additionally, there is also data indicated that this patient cohort may also benefit from neoadjuvant treatment strategies [44, 45]. In 2021, Wu et al. showed that neoadjuvant Intensity-Modulated Radiotherapy plus surgery is effective and well-tolerated in patients with centrally located HCC [44]. In a randomized, multicenter controlled trial, Wei et al. showed that three-dimensional conformal neoadjuvant radiotherapy provided significantly better postoperative survival outcomes than surgery alone for resectable HCC with a portal vein thrombosis [45].

There are some limitations to our study. First, it is a retrospective, prognostic study with a potential selection and reporting bias. Second, the identified cut-off values of DTV in our study were not tested in a separate test cohort to verify the optimal cut-off values. Lastly, our results were from a single-center study, which might not be applicable to other population with different etiologies of liver disease. Further confirmation of the results in a prospective, multicenter setting would be needed.

In conclusion, DTV is a promising predictor of RFS and OS in HCC. This study provides novel insights into the prognostic role of DTV in patients with HCC.

## Supplementary Information

Below is the link to the electronic supplementary material.

ESM 1 (36.6 KB)

## Data Availability

The data that support the findings of this study are available on request from the corresponding author.

## Abbreviations

AUC: area under the curve
CT: computed tomography
DTV: distance of the tumor from the main hepatic vessels
HCC: hepatocellular carcinoma
MVI: microvascular invasion
MRI: magnetic resonance imaging
ROC: Receiver operating characteristics

## References

[1] Sung H et al (2021) Global Cancer statistics 2020: GLOBOCAN estimates of incidence and Mortality Worldwide for 36 cancers in 185 countries. CA Cancer J Clin 71(3):209–249 33538338 10.3322/caac.21660

[2] Ghouri YA, Mian I, Rowe JH (2017) Review of hepatocellular carcinoma: epidemiology, etiology, and carcinogenesis. J Carcinog 16:1 28694740 10.4103/jcar.JCar_9_16 PMC5490340

[3] EASL (2018) Clinical Practice guidelines: management of hepatocellular carcinoma. J Hepatol 69(1):182–236. 10.1016/j.jhep.2018.03.019 10.1016/j.jhep.2018.03.019 29628281

[4] Zhu Y et al (2020) Factors influencing early recurrence of hepatocellular carcinoma after curative resection. J Int Med Res 48(8):300060520945552 33106072 10.1177/0300060520945552 PMC7780562

[5] Jung SM et al (2019) Characteristics of early recurrence after curative liver resection for Solitary Hepatocellular Carcinoma. J Gastrointest Surg 23(2):304–311 30215196 10.1007/s11605-018-3927-2

[6] Lee HA et al (2021) Change in the recurrence pattern and predictors over Time after Complete Cure of Hepatocellular Carcinoma. Gut Liver 15(3):420–429 32839362 10.5009/gnl20101 PMC8129665

[7] Xing H et al (2020) Defining and predicting early recurrence after liver resection of hepatocellular carcinoma: a multi-institutional study. HPB (Oxford) 22(5):677–689 31607637 10.1016/j.hpb.2019.09.006

[8] Kumar S et al (2020) Isolation of Tumor cells based on their Distance from Blood vessels. Bio Protoc 10(10):e3628. 10.21769/BioProtoc.3628 10.21769/BioProtoc.3628 PMC7842338 33659301

[9] Kumar S et al (2019) Intra-tumoral metabolic zonation and resultant phenotypic diversification are dictated by blood Vessel Proximity. Cell Metab 30(1):201–211e6 31056286 10.1016/j.cmet.2019.04.003

[10] Birgin E et al (2022) Minimally invasive mesohepatectomy for centrally located liver lesions-a case series. Surg Endosc 36(12):8935–8942 35668311 10.1007/s00464-022-09342-3 PMC9652264

[11] Birgin E et al (2022) A postresection perfusion deficit in the right colon is an independent predictor of perioperative outcome after major hepatectomy. J Hepatobiliary Pancreat Sci 29(7):785–797 34856068 10.1002/jhbp.1089

[12] Birgin E et al (2024) Robotic or laparoscopic repeat hepatectomy after open hepatectomy: a cohort study. Surg Endosc 38(3):1296–1305 38102396 10.1007/s00464-023-10645-2

[13] von Elm E et al (2008) The strengthening the reporting of Observational studies in Epidemiology (STROBE) statement: guidelines for reporting observational studies. J Clin Epidemiol 61(4):344–349 18313558 10.1016/j.jclinepi.2007.11.008

[14] Rahbari NN et al (2011) Posthepatectomy liver failure: a definition and grading by the International Study Group of Liver surgery (ISGLS). Surgery 149(5):713–724 21236455 10.1016/j.surg.2010.10.001

[15] Rahbari NN et al (2011) Post-hepatectomy haemorrhage: a definition and grading by the International Study Group of Liver Surgery (ISGLS)*.* HPB (Oxford) 13(8): 528 – 35. 10.1111/j.1477-2574.2011.00319.x 10.1111/j.1477-2574.2011.00319.x PMC3163274 21762295

[16] Birgin E, Reißfelder C, Rahbari NN (2024) Robot with the scissorhands: scissor hepatectomy for parenchymal transection in robotic liver resection. J Gastrointest Surg 28(1):99–101 38353085 10.1016/j.gassur.2023.11.018

[17] Wakabayashi G et al (2022) The Tokyo 2020 terminology of liver anatomy and resections: updates of the Brisbane 2000 system. J Hepatobiliary Pancreat Sci 29(1):6–15 34866349 10.1002/jhbp.1091

[18] Jungermann K, Kietzmann T (1996) Zonation of parenchymal and nonparenchymal metabolism in liver. Annu Rev Nutr 16:179–203 8839925 10.1146/annurev.nu.16.070196.001143

[19] Sasse D, Spornitz UM, Maly IP (1992) Liver Archit Enzyme 46(1–3):8–32 10.1159/000468776 1289084

[20] Kietzmann T (2017) Metabolic zonation of the liver: the oxygen gradient revisited. Redox Biol 11:622–630 28126520 10.1016/j.redox.2017.01.012 PMC5257182

[21] Kietzmann T et al (2006) Oxygen: modulator of physiological and pathophysiological processes in the liver. Z Gastroenterol 44(01):67–76 16397842 10.1055/s-2005-858987

[22] Greger R, Bleich M (1996) Normal values for physiological parameters. Comprehensive Human physiology: from Cellular mechanisms to Integration. R. Greger and U. Windhorst (eds) Springer, Berlin Heidelberg, p. 2427–2449

[23] Quistorff B, Dich J, Grunnet N (1986) Periportal and perivenous hepatocytes retain their zonal characteristics in primary culture. Biochem Biophys Res Commun 139(3):1055–1061 3021146 10.1016/s0006-291x(86)80284-4

[24] García-Vilas JA, Medina M (2018) Updates on the hepatocyte growth factor/c-Met axis in hepatocellular carcinoma and its therapeutic implications. World J Gastroenterol 24(33):3695–3708 30197476 10.3748/wjg.v24.i33.3695 PMC6127652

[25] Xie Q et al (2001) SF/HGF-c-Met autocrine and paracrine promote metastasis of hepatocellular carcinoma. World J Gastroenterol 7(6):816–820 11854908 10.3748/wjg.v7.i6.816 PMC4695601

[26] Trusolino L, Bertotti A, Comoglio PM (2010) MET signalling: principles and functions in development, organ regeneration and cancer. Nat Rev Mol Cell Biol 11(12):834–848 21102609 10.1038/nrm3012

[27] Bozkaya G et al (2012) Cooperative interaction of MUC1 with the HGF/c-Met pathway during hepatocarcinogenesis. Mol Cancer 11:64 22962849 10.1186/1476-4598-11-64 PMC3542123

[28] Morse MA et al (2019) The role of Angiogenesis in Hepatocellular Carcinoma. Clin Cancer Res 25(3):912–920 30274981 10.1158/1078-0432.CCR-18-1254

[29] Cramer T, Vaupel P (2022) Severe hypoxia is a typical characteristic of human hepatocellular carcinoma: scientific fact or fallacy? J Hepatol 76(4):975–980 34990751 10.1016/j.jhep.2021.12.028

[30] Xiong XX et al (2017) Advances in hypoxia-mediated mechanisms in Hepatocellular Carcinoma. Mol Pharmacol 92(3):246–255 28242743 10.1124/mol.116.107706

[31] Xing L et al (2023) ROS in hepatocellular carcinoma: what we know. Arch Biochem Biophys 744:109699. 10.1016/j.abb.2023.109699 10.1016/j.abb.2023.109699 37499994

[32] Li Y et al (2023) Insights into the role of oxidative stress in Hepatocellular Carcinoma Development. FBL 28(11). 10.31083/j.fbl2811286 10.31083/j.fbl2811286 38062825

[33] Hsu SY, Liang SG, Hsueh AJ (1998) Characterization of two LGR genes homologous to gonadotropin and thyrotropin receptors with extracellular leucine-rich repeats and a G protein-coupled, seven-transmembrane region. Mol Endocrinol 12(12):1830–1845 9849958 10.1210/mend.12.12.0211

[34] Liu J et al (2017) LGR5 promotes hepatocellular carcinoma metastasis through inducting epithelial-mesenchymal transition. Oncotarget 8(31):50896–50903 28881613 10.18632/oncotarget.15143 PMC5584214

[35] Gebhardt R, Matz-Soja M (2014) Liver zonation: novel aspects of its regulation and its impact on homeostasis. World J Gastroenterol 20(26):8491–8504 25024605 10.3748/wjg.v20.i26.8491 PMC4093700

[36] Della Corte CM et al (2017) Implication of the hedgehog pathway in hepatocellular carcinoma. World J Gastroenterol 23(24):4330–4340 28706416 10.3748/wjg.v23.i24.4330 PMC5487497

[37] Frenkel NC et al (2020) Liver lymphatic drainage patterns follow segmental anatomy in a murine model. Sci Rep 10(1):21808. 10.1038/s41598-020-78727-y 10.1038/s41598-020-78727-y PMC7732834 33311587

[38] Tanaka M, Iwakiri Y (2016) The hepatic lymphatic vascular system: structure, function, markers, and Lymphangiogenesis. Cell Mol Gastroenterol Hepatol 2(6):733–749 28105461 10.1016/j.jcmgh.2016.09.002 PMC5240041

[39] Sneag DB et al (2011) Extrahepatic spread of hepatocellular carcinoma: spectrum of imaging findings. AJR Am J Roentgenol 197(4):W658–W664 21940537 10.2214/AJR.10.6402

[40] Heimbach JK et al (2018) AASLD guidelines for the treatment of hepatocellular carcinoma. Hepatology 67(1):358–380 28130846 10.1002/hep.29086

[41] Vogel A, Meyer T, Saborowski A (2023) IMbrave050: the first step towards adjuvant therapy in hepatocellular carcinoma. Lancet 402(10415):1806–1807 37883983 10.1016/S0140-6736(23)01962-1

[42] Sidaway P (2024) Adjuvant sintilimab effective in high-risk HCC. Nat Rev Clin Oncol 21(3):168 38316989 10.1038/s41571-024-00865-3

[43] Wang K et al (2024) Adjuvant sintilimab in resected high-risk hepatocellular carcinoma: a randomized, controlled, phase 2 trial. Nat Med 30(3):708–715 38242982 10.1038/s41591-023-02786-7

[44] Wu F et al (2022) Phase 2 evaluation of Neoadjuvant intensity-modulated Radiotherapy in centrally located Hepatocellular Carcinoma: a Nonrandomized Controlled Trial. JAMA Surg 157(12):1089–1096 36197682 10.1001/jamasurg.2022.4702 PMC9535533

[45] Wei X et al (2019) Neoadjuvant three-Dimensional Conformal Radiotherapy for Resectable Hepatocellular Carcinoma with Portal Vein Tumor Thrombus: a randomized, Open-Label, Multicenter Controlled Study. J Clin Oncol 37(24):2141–2151 31283409 10.1200/JCO.18.02184 PMC6698917

