# Supplementary material for: The tumor distance to the main hepatic vessels is a predictor of recurrence-free survival and overall survival in hepatocellular cancer
Source: Langenbecks Arch Surg. 2025 Jan 9;410(1):31. doi: 10.1007/s00423-024-03565-9 (PMC11717872; doi:10.1007/s00423-024-03565-9)
Supplement: Supplementary file 1 — ESM 1 (36.6 KB) [file 423_2024_3565_MOESM1_ESM.docx]

**Supplemental Material**

**Table S1: Operative characteristics and postoperative outcomes of the study population**

| **Characteristics** | **Total (n=84)** |
| --- | --- |
| **Extent of resection** |  |
| Major hepatectomy | 21 (25) |
| Minor hepatectomy | 63 (75) |
| **Surgical approach** |  |
| Open | 12 (14) |
| Laparoscopic | 61 (72) |
| Robotic | 11 (13) |
| **Surgical procedure** |  |
| Non-anatomic resections | 10 (12) |
| Right (extended) hepatectomy | 10 (12) |
| Left (extended) hepatectomy | 11 (13) |
| Left lateral sectionectomy | 10 (12) |
| Right anterior sectionectomy | 0 (0) |
| Right posterior sectionectomy | 4 (5) |
| Other anatomical segmentectomies | 39 (46) |
| Monosegmentectomy | 16 (19) |
| Bisegmentectomy | 19 (23) |
| Trisegmentectomy | 4 (5) |
| **Operative time, min^a^** | 256 (181–342) |
| **Pringle maneuver** | 51 (61) |
| Duration, min^a^ | 40 (24–68) |
| **IVC clamping** | 5 (6) |
| **Blood loss, ml^a^** | 600 (250–1250) |
| **Resections margins** |  |
| R0 | 78 (93) |
| R1 | 6 (7) |
| **Microvascular invasion** | 21 (25) |
| **Length of stay, d^a^** | 7 (3-89) |
| **Postoperative complications^b^** |  |
| Grade I | 16 (19) |
| Grade II | 14 (17) |
| Grade III | 7 (8) |
| Grade IV | 5 (6) |
| Grade V | 6 (7) |
| **Type of complications** |  |
| Wound infection | 5 (6) |
| Burst abdomen | 2 (2) |
| Pleural effusion with atelectasis | 13 (15) |
| Pulmonary embolism | 2 (2) |
| Posthepatectomy hemorrhage^c^ | 5 (6) |
| Posthepatectomy bile leakage^d^ | 5 (6) |
| Posthepatectomy liver failure^e^ | 10 (12) |

*IVC* infrahepatic vena cava, *R0* no residual tumor, *R1* microscopic residual tumor

^a^Values are median (interquartile range)

^b^Clavien-Dindo classification

^c^Grad A International study group of liver surgery (n=4), Grad B International study group of liver surgery (n=1)

^d^Grad A International study group of liver surgery (n=3), Grad B International study group of liver surgery (n=2)
^e^Grad A International study group of liver surgery (n=8), Grad B International study group of liver surgery (n=2)

**Table S2: Cox proportional hazard analysis of predictive factors for recurrence-free survival**

| **Characteristics** | **Univariable analysis** | | |  | **Multivariable analysis** | | |
| --- | --- | --- | --- | --- | --- | --- | --- |
|  | **HR** | **95%CI** | ***p*-value** |  | **HR** | **95%CI** | ***p*-value** |
| **Age (years)^a^** | 1.09 | 0.98–1.21 | 0.11 |  |  |  |  |
| **BMI (kg/m²)^a^** | 0.96 | 0.78–1.10 | 0.74 |  |  |  |  |
| **Sex ratio (Male: Female)** | 0.89 | 0.36–2.18 | 0.79 |  |  |  |  |
| **ASA** |  |  |  |  |  |  |  |
| I | Ref | Ref | Ref |  |  |  |  |
| II | 1.28 | 0.17–9.82 | 0.81 |  |  |  |  |
| III | 1.29 | 0.18–9.83 | 0.80 |  |  |  |  |
| IV | 1.53 | 0.10–24.27 | 0.76 |  |  |  |  |
| **Cardiovascular comorbidities (Yes vs. no)** | 1.53 | 0.62-3.79 | 0.36 |  |  |  |  |
| **Diabetes mellitus (Yes vs. no)** | 1.06 | 0.50–2.25 | 0.88 |  |  |  |  |
| **Pulmonary comorbidities (Yes vs. no)** | 1.48 | 0.68–3.19 | 0.35 |  |  |  |  |
| **Liver cirrhosis (Yes vs. no)** | 0.64 | 0.08–5.18 | 0.68 |  |  |  |  |
| **Child-Pugh classification** |  |  |  |  |  |  |  |
| Child A | Ref | Ref | Ref |  |  |  |  |
| Child B | 0.50 | 0.23–1.10 | 0.08 |  |  |  |  |
| **Etiology of cirrhosis** |  |  |  |  |  |  |  |
| Alcohol | Ref | Ref | Ref |  |  |  |  |
| Viral | 1.13 | 0.25–5.11 | 0.87 |  |  |  |  |
| Hepatitis B | Ref | Ref | Ref |  |  |  |  |
| Hepatitis C | 1.54 | 0.14–17.10 | 0.72 |  |  |  |  |
| MASLD | 2.62 | 0.48–14.08 | 0.26 |  |  |  |  |
| **Preoperative laboratory tests** |  |  |  |  |  |  |  |
| Albumin (g/l) | 1.01 | 0.94–1.09 | 0.76 |  |  |  |  |
| Bilirubin (mg/dl) | 0.76 | 0.20–2.86 | 0.67 |  |  |  |  |
| INR | 0.16 | 0.00–21.13 | 0.46 |  |  |  |  |
| Platelets (×10^9^/l) | 1.00 | 1.00–1.01 | 0.27 |  |  |  |  |
| AP (U/l) | 1.00 | 0.99–1.01 | 0.58 |  |  |  |  |
| gGT (U/l) | 1.00 | 1.00–1.00 | 0.43 |  |  |  |  |
| AST (U/l) | 1.00 | 0.99–1.01 | 0.41 |  |  |  |  |
| ALT (U/l) | 1.00 | 0.99–1.01 | 0.53 |  |  |  |  |
| **Previous treatment** |  |  |  |  |  |  |  |
| Previous hepatic resection | 1.30 | 0.50–3.42 | 0.59 |  |  |  |  |
| Previous locoregional therapy | 0.54 | 0.19–4.68 | 0.45 |  |  |  |  |
| Previous Y90 treatment | 0.69 | 0.15–5.26 | 0.62 |  |  |  |  |
| Previous systemic treatment | 0.69 | 0.15–5.26 | 0.62 |  |  |  |  |
| **Radiological characteristics** |  |  |  |  |  |  |  |
| DTV, mm | 0.91 | 0.87–0.95 | <0.001 |  | 0.94 | 0.89–0.99 | 0.03 |
| Number of lesions | 0.88 | 0.55–1.41 | 0.59 |  |  |  |  |
| Tumor size, mm | 1.01 | 1.00–1.02 | <0.001 |  | 1.01 | 0.99–1.02 | 0.02 |
| Number of infiltrated segments | 1.16 | 0.91–1.51 | 0.25 |  |  |  |  |

| **Characteristics** | **Univariable analysis** | | |  | **Multivariable analysis** | | |
| --- | --- | --- | --- | --- | --- | --- | --- |
|  | **HR** | **95%CI** | ***p*-value** |  | **HR** | **95%CI** | ***p*-value** |
| **Extent of resection** |  |  |  |  |  |  |  |
| Major hepatectomy | Ref | Ref | Ref |  |  |  |  |
| Minor hepatectomy | 0.96 | 0.43–2.18 | 0.92 |  |  |  |  |
| **Surgical approach** |  |  |  |  |  |  |  |
| Open | Ref | Ref | Ref |  |  |  |  |
| Laparoscopic | 0.68 | 0.23–1.28 | 0.92 |  |  |  |  |
| Robotic | 0.47 | 0.07–6.49 | 0.69 |  |  |  |  |
| **Surgical procedure** |  |  |  |  |  |  |  |
| Non-anatomic resections | Ref | Ref | Ref |  |  |  |  |
| Right (extended) hepatectomy | 0.00 | 0.00–Inf | 0.99 |  |  |  |  |
| Left (extended) hepatectomy | 0.9 | 0.27–2.95 | 0.86 |  |  |  |  |
| Left lateral sectionectomy | 0.36 | 0.10–1.37 | 0.13 |  |  |  |  |
| Right posterior sectionectomy | 1.35 | 0.25–7.34 | 0.73 |  |  |  |  |
| Other anatomical segmentectomies | 0.35 | 0.12–1.01 | 0.06 |  |  |  |  |
| **Operative time, min** | 1.00 | 1.00–1.00 | 0.42 |  |  |  |  |
| **Pringle maneuver (Yes vs. no)** | 0.65 | 0.27–1.35 | 0.35 |  |  |  |  |
| Duration, min | 0.97 | 0.98–1.01 | 0.68 |  |  |  |  |
| **IVC clamping** | 1.01 | 0.96–1.04 | 0.87 |  |  |  |  |
| **Blood loss, ml** | 1.00 | 1.00–1.00 | 0.14 |  |  |  |  |
| **Resections margins** |  |  |  |  |  |  |  |
| R0 | Ref | Ref | Ref |  | Ref | Ref | Ref |
| R1 | 3.41 | 1.08–9.26 | 0.03 |  | 1.99 | 0.38–10.31 | 0.41 |
| **T classification** |  |  |  |  |  |  |  |
| 1 | Ref | Ref | Ref |  |  |  |  |
| 2 | 0.82 | 0.46–1.46 | 0.50 |  |  |  |  |
| 3 | 0.59 | 0.77–16.12 | 0.33 |  |  |  |  |
| 4 | 0.00 | 0.00–Inf | 0.99 |  |  |  |  |
| **Nodal status** |  |  |  |  |  |  |  |
| 0 | Ref | Ref | Ref |  |  |  |  |
| 1 | 0.06 | 0.33–1.07 | 0.08 |  |  |  |  |
| **M status** |  |  |  |  |  |  |  |
| 0 | Ref | Ref | Ref |  |  |  |  |
| 1 | 0.93 | 0.13–6.80 | 0.95 |  |  |  |  |
| **Grading** |  |  |  |  |  |  |  |
| 1 | Ref | Ref | Ref |  | Ref | Ref | Ref |
| 2 | 3.41 | 1.25–9.32 | 0.017 |  | 2.17 | 0.75–6.28 | 0.15 |
| 3 | 11.5 | 3.24–40.79 | < 0.001 |  | 4.15 | 0.91–18.98 | 0.06 |
| **Microvascular invasion** |  |  |  |  |  |  |  |
| 0 | Ref | Ref | Ref |  | Ref | Ref | Ref |
| 1 | 4.53 | 2.13–9.61 | < 0.001 |  | 2.99 | 1.18–7.55 | 0.02 |

| **Characteristics** | **Univariable analysis** | | |  | **Multivariable analysis** | | |
| --- | --- | --- | --- | --- | --- | --- | --- |
|  | **HR** | **95%CI** | ***p*-value** |  | **HR** | **95%CI** | ***p*-value** |
| **Length of stay, d** | 1.01 | 0.95–1.07 | 0.71 |  |  |  |  |
| **Postoperative complications^a^** |  |  |  |  |  |  |  |
| Grade I | Ref | Ref | Ref |  |  |  |  |
| Grade II | 2.49 | 0.88–7.03 | 0.08 |  |  |  |  |
| Grade III | 0.89 | 0.11–7.61 | 0.98 |  |  |  |  |
| Grade IV | 4.16 | 0.80–21.70 | 0.09 |  |  |  |  |
| Grade V | 0.00 | 0.00–Inf | 0.99 |  |  |  |  |
| **Type of complications** |  |  |  |  |  |  |  |
| Wound infection | 1.73 | 0.11–27.89 | 0.69 |  |  |  |  |
| Burst abdomen | 0.00 | 0.00–Inf | 1.00 |  |  |  |  |
| Pleural effusion with atelectasis | 0.86 | 0.11–6.68 | 0.88 |  |  |  |  |
| Pulmonary embolism | 58x10^8^ | 0.00–Inf | 1.00 |  |  |  |  |
| Posthepatectomy hemorrhage | 2.06 | 0.48–15.31 | 0.33 |  |  |  |  |
| Posthepatectomy bile leakage | 0.00 | 0.00–Inf | 0.98 |  |  |  |  |
| Posthepatectomy liver failure | 0.27 | 0.04–1.99 | 0.19 |  |  |  |  |

*BMI* body mass index, *ASA* American Society of Anesthesiologists*, HR* hazard ratio*, CI* confidence interval*, NA* not available,

*Ref* reference, *INR* international normalized ratio, *AP* alkaline phosphatase, *gGT* gamma-glutamyltransferase, *AST* aspartate

aminotransferase, *ALT* alanine aminotransferase, *DTV* distance of the tumor from the main hepatic vessels, *IVC* infrahepatic

vena cava, *MVI* microvascular invasion, *R0* no residual tumor, *R1* microscopic residual tumor, *X* missing values, *Inf* infinity

^a^Clavien-Dindo classification
